# Modulation of Antioxidant Defense, Immune Response, and Growth Performance by Inclusion of Propolis and Bee Pollen into Broiler Diets

**DOI:** 10.3390/ani12131658

**Published:** 2022-06-28

**Authors:** Saad N. AL-Kahtani, Abdulaziz A. Alaqil, Ahmed O. Abbas

**Affiliations:** 1 Department of Arid Land Agriculture, College of Agricultural and Food Sciences, King Faisal University, P.O. Box 420, Al-Ahsa 31982, Saudi Arabia; salkahtani@kfu.edu.sa; 2 Department of Animal and Fish Production, College of Agricultural and Food Sciences, King Faisal University, P.O. Box 420, Al-Ahsa 31982, Saudi Arabia; 3 Department of Animal Production, Faculty of Agriculture, Cairo University, 7 Gamma St., Giza 12613, Egypt

**Keywords:** propolis, bee pollen, productive performance, antioxidants, immune response, broilers

## Abstract

**Simple Summary:**

Broiler meat provides a considerable nutritional source of animal protein in the human diet. However, the intensive and accelerating growth in broiler breeding programs requires a continuous or intermittent use of antibiotics to improve the health and performance of broilers. Antibiotic resistance and residues problems cause a general limitation on the use of antibiotics in poultry production worldwide, and hence, prompt researchers and feed companies to find natural alternatives. In the present study, we investigated the possible impact of propolis (PR) and bee pollen (BP) in nutritional strategies on the performance and immunity of broiler chickens. The obtained results display the ability of PR and BP contained within the broiler diets to enhance the antioxidant defense system and improve several immunological parameters. These beneficial effects coincided with an increase in the growth performance of broilers. Thus, supplementation of PR and BP separately or in combination could be recommended into broiler diets for their positive impacts as natural products on the performance and health of broilers.

**Abstract:**

(1) Background: Propolis and bee pollen have natural bioactive compounds that may support the performance and immunological response of broilers. (2) Methods: The study included 300 1 d old Cobb-500 broiler chicks. Starting from 22–42 d of age, chicks were divided according to a 2 × 2 factorial design into one of the four treatment groups (5 replicates × 15 chicks per replicate); a basal diet without supplementation (CONT) or supplemented with 1 g/kg of propolis (PR) or bee pollen (BP) separately or in an even combination (PR + BP). (3) Results: A significant (*p* < 0.05) increase was obtained in the body-weight gain of broilers treated with PR, BP, and PR + BP compared to the CONT. The total antioxidant capacity and superoxide dismutase were highly (*p* < 0.05) activated in all treated groups compared to the CONT. Immunological parameters, especially the leukocyte cell viability, T- and B-lymphocyte proliferation, immunoglobulins (IgA and IgM), antibody titers, and wattle-swelling test were significantly (*p* < 0.05) enhanced in the treated broilers with PR and/or BP compared to the CONT. (4) Conclusions: The dietary supplementation of PR and/or BP could be beneficial for broiler growth through maximizing the antioxidant- and immune-system defenses.

## 1. Introduction

The intensive and rapid production in broiler selection programs require continuous or intermittent courses of antibiotic therapy to improve broiler well-being and performance. However, antibiotic resistance and residues problems lead to a general limitation on the use of antibiotics in poultry production worldwide, and prompt researchers and feed companies to seek natural alternatives [1]. Recently, the natural products have been widely considered in nutritional strategies for optimizing the health and performance of poultry during intensive production. Among the regarded candidates of natural products are those produced by honey-bee workers, such as propolis (PR) and bee pollen (BP) [2]. 

Propolis consists of resinous substances from various plants in addition to essential oils and waxes gathered by the bees [3], while BP consists of pollen grains mixed with nectar and the hypopharyngeal-glands secretion of the bees [4]. Both products are considered to be feed supplements in animal nutrition due to their abundant sources of nutrients [5,6], flavonoids [7,8], antioxidants [4,9], digestive enzymes [10,11], and antimicrobial compounds [12,13].

The beneficial effects of PR and BP on poultry production and health have been documented in research. It was reported that dietary PR supplementation reduced the oxidative stress induced by paraquat herbicides in turkey [14] or by heat stress in Japanese quail [15]. Bee pollen supplementation into broiler diets promotes some immunological traits, such as increasing leukocytes, decreasing heterophil/lymphocyte ratio, speeding the antibody production, and reinforcing the immune-organs formation [16,17]. In addition, broiler growth aspects were enhanced by dietary PR and/or BP supplementation through morphological and bacterial regulation in the gastrointestinal tract [13]. Further studies concluded that honeybee products, including PR and BP, improve the growth performance and immune functions in Japanese quail [18]. However, the information available in the literature about the addition of bee products into broiler diets remain scant, especially regarding the physiological mechanism of action such as immunological and antioxidant status. The objective of this study was to highlight the possible impact of PR and BP inclusion, alone or together, into broiler diets on their performance along with the antioxidant and immunological defense system. 

## 2. Materials and Methods

### 2.1. PR and BP Preparation and Analysis

The PR and BP were obtained from a collection of beehives situated in the Agricultural and Veterinary Research Station, King Faisal University, Saudi Arabia. Propolis was obtained in the form of yellow-brown powder, while dry BP was obtained as small yellow pellets. Samples of PR and BP were subjected to a chemical analysis based on the methods of International AOAC [19]. The total phenolic contents of both PR and BP were estimated according to the Folin–Ciocalteu colorimetric methods [20], considering gallic acid as the standard and obtaining the optical density by a CE1010-Spectrophotometer (Cecil Instruments Limited, Cambridge, UK) at 765 nm. The total flavonoids in PR and BP were also determined according to the aluminum calorimetric methods [21], considering catechol as the standard and measuring the absorbance at 435 nm by the spectrophotometer. In addition, the scavenging activities of PR and BP samples against 2,2-diphenyl-1-picrylhydrazyl (DPPH)-free radicals were measured by the spectrophotometer at 520 nm [22]. Table 1 represents the chemical characteristics of the PR and BP used in the experiment.

### 2.2. Birds and Treatments

A total of 300 male broiler chicks (Cobb500™) were obtained from a local hatchery at one day of age and raised in an open-system floor house. During the experiment, all the chicks were maintained within the same optimum conditions of temperature, humidity, and lighting as recommended by the manufacturer’s guideline of Cobb-500 broiler management (available at: https://www.cobb-vantress.com/en_US/products/cobb500/; accessed on 1 March 2022). According to the guidelines, basal diets of soybean–corn mixture were formulated to meet the standard requirements of Cobb-500 broilers (Table 2). Birds were given unlimited access to food and water during the experiment. 

From 22–42 d of age, birds were randomly assigned into 4 treatment groups according to a 2 × 2 factorial design with five replicates of 15 birds each (75 chicks per group). The birds in each replicate were raised on 5 cm-deep litter of wood-shavings in a floor yard area of 1.35 × 1.35 m^2^. The first group served as a control and was fed on a basal diet without supplementation (CONT). The other experimental groups were fed on a basal diet supplemented with either 1 g/kg propolis (PR group), 1 g/kg bee pollen (BP group), or an even mixture of PR and BP at 1 g/kg each (PR + BP group), respectively. Both PR and BP were crushed using a grinder (Moulinex Type LM201, Mayenne, France) and mixed daily with the basal diet before they were introduced to the broilers. The productive performance of chicks was evaluated during the experimental period length of 22–42 d of age. Furthermore, blood samples were taken from birds when the treatments ended at 42 days of age for the purpose of studying some antioxidant indicators and immunological response, as posteriorly described in detail. 

### 2.3. Productive Performance

Individual body weights in each group were recorded at days 22 and 42 of age to determine the initial body weight (IBW), final body weight (FBW), and body weight gain (BWG) for each experimental group. Feed intake (FI) was calculated by taking the leftover feed from the total amount of feed that was given for each replicate in the treatment group. The feed conversion ratio (FCR) was then determined for each replicate in the treatment group based on FI per unit of BWG. 

### 2.4. Antioxidant Indicators

As soon as the treatments were over, two blood samples from the brachial vein were taken for each replicate per experimental group (n = 10) and immediately transferred into heparinized tubes. Plasma was separated by centrifuging the blood samples for 10 min at 2000× *g* at 4 °C and stored to figure out the antioxidant indicators. The total antioxidant capacity (TAC), total superoxide dismutase (T-SOD), and catalase (CAT) assays were performed using an automated microplate scanner and the available colorimetric kits (MBS2540515, MBS2563691, and MBS2540413, respectively; MyBioSource, San Diego, CA, USA) according to the manufacturer’s instructions. Table S1 summarizes the detection limits, sensitivity, intra-assay CV%, and inter-assay CV% for each assay. 

### 2.5. Immunological Parameters

#### 2.5.1. Leukocyte’s Count, Differentiation, and Viability

Two blood samples per replication in each experimental group (n = 10) were taken at the conclusion of the treatments (42 d of age) and relocated into heparinized tubes. Ten µL of the fresh sample was diluted with 490 µL brilliant cresyl blue stain solution. A drop of the mixture was mounted on a hemocytometer slide, and the total white blood cells (TWBC) count was then detected under a microscope at 200× magnification [23]. Another 10 µL of the blood sample was smeared on a glass slide, then fixed and stained using Hema-3 solutions (Fisher Scientific, Pittsburg, PA, USA). Differentiation of approximately 200 leukocytes was performed under a microscope at 1000× magnification with oil immersion, and the heterophil-to-lymphocyte (H/L) ratio was then detected [24]. 

The remaining blood samples were allocated to determine leucocyte cell viability (LCV) according to the methods described by Abbas et al. [25]. First, peripheral blood mononuclear cells (PBMC) were separated by centrifuging the blood samples with Histopaque-1077 medium (Sigma Chemical Co., St. Louis, MO, USA) at 1030× *g* for 20 min at 4 °C. The PBMCs were washed twice using RPMI-1640 culture medium (Thermo Fisher Scientific, Waltham, MA, USA), then resuspended with phosphate-buffered saline (PBS) (pH 7.2). Thereafter, 100 µL of cell suspension were pipetted with 25 µL of MTT solution (5 mg of tetrazolium salt; MTT, Serva, Heidelberg, Germany; dissolved in 1 mL of AIM-V medium; Thermofisher) in a 96-well plate. The plates were incubated for 4 h at 37 °C, then centrifuged at 600× *g* for 10 min. The incubation medium was discarded, and each well was refilled with 100 µL of acidified isopropyl alcohol solution (0.04 N HCl). Finally, the absorbance of formazan was measured at 570 nm using an automated ELISA reader (Bio-Rad Laboratories Inc., Hercules, CA, USA).

#### 2.5.2. Lymphocyte Proliferation

Proliferation of T- and B-lymphocyte cells was evaluated in blood samples obtained from 2 broilers per replicate (n = 10 per treatment group) according to the methodology described by Alaqil et al. [26]. The protocol, in brief, started with the isolation of the PBMCs, washing twice, and resuspending in RPMI-1640 medium as previously stated. The viable lymphocytes in each sample were plated in triplicates at a constant concentration of 1 × 10^6^ cells/mL in a 96-well plate. The experimental wells were supplemented with 50 μL of 5% Concanavalin-A mitogen or 1% Lipopolysaccharide to stimulate T- or B-lymphocytes, respectively, whereas the control wells were filled with 50 μL RPMI medium. The samples were incubated for 48 h at 42 °C, 5% CO_2,_ and saturated humidity, then appended with 15 μL of MTT solution and incubated again for 4 h, and finally complemented with 100 μL of 10% sodium dodecyl sulfates in 0.04 M HCl. The optical density at 570 nm (OD570) was recorded for the experimental wells against the control wells using an automated ELISA. Stimulation index (SI) of T- and B-lymphocytes was computed as the OD570 ratio for stimulated to unstimulated cells in each sample. 

#### 2.5.3. Immunoglobulins Assay

Blood samples were collected into heparinized tubes from 2 chicks per replicate in each treatment group (n = 10). The plasma was separated by centrifuging at 2000× *g* for 10 min at 4 °C then kept at −20 °C to be used in immunoglobulin (Ig) assay. The IgA, IgM, and IgG were analyzed in accordance with the manufacturer instructions of commercial ELISA kits specific for chickens (MBS564152, MBS706158 and MBS260043, respectively; MyBioSource). In brief, 100 μL of the diluted samples (1:5000 in the sample-conjugate diluent) or standards were pipetted (in duplicate) into predesignated wells in a microtiter plate. After incubation, the plates were washed twice, and the contents were removed by sharp striking on an absorbent paper. The plate was then incubated in the dark after the addition of 100 μL of an appropriate dilution of enzyme–antibody conjugate. The contents were removed after being washed three times, then 100 μL of Chromogen-substrate solution were pipetted to each well and incubated in the dark. Finally, the reaction was stopped by the addition of 100 μL sulfuric acid (0.3 M) and the absorbance was read at 450 nm using ELISA microplate-reading scanner. The IgA, IgM, and IgG levels were computed using a 4-parameter logistic curve fit generated from the chicken reference serum absorbance. The specifications of the immunoglobulin ELISA assays are summarized in Table S2.

#### 2.5.4. Humoral and Cellular Immunity Assay

The sheep red blood cells antibody (SRBC-AB) titer was evaluated to point out the humoral immunity of broilers in this study. One week before the end of the experiment (at 35 d of age), 2 birds per replicate per treatment group (n = 10) received an IV injection with 1 mL of 5% SRBC. After that, blood samples were taken from the birds, allowed for clotting at RT for 2 h, then centrifuged at 4 °C for 10 min at 400× *g* to separate the sera. Serial doubling dilutions of sera samples (25 μL each) were pipetted in a 96-well plate, and 25 μL of 2% SRBC solution was added to each dilution. The plates were gently vortexed and kept overnight for agglutination at RT. The antibody titer was calculated as log_2_ value of the inverse of the last dilution with positive agglutination in the well’s bottom [27].

The cellular immunity of broilers was assessed using procedures outlined in a prior study [28]. At 42 d of age, 2 birds per replication in each treatment group (n = 10) were assigned for the test. Briefly, birds were intradermally injected with 0.1 mL sterile PBS supplemented with 0.5 mg mitogenic phytohemagglutinin (PHA) (Thermo Fisher Scientific) in a marked area of the wattle. Twenty-four hours later, the increase in the wattle thickness was measured as a positive reaction to the PHA-wattle immune test. 

### 2.6. Statistical Analysis

Data for all variables were analyzed using two-way analysis of variance (ANOVA) and explored with a General Linear Model (GLM) procedure of SPSS software (version 22.0; IBM Corp., Armonk, NY, USA, 2013). The main factors were propolis supplementation (−PR versus + PR) and bee pollen supplementation (−BP versus + BP). The interaction between the two main factors (PR × BP) was tested and represented as CONT, PR, BP, and PR + BP, respectively. The experimental unit was considered to be the number of observations per treatment group for each test performed (n = 5 for the productive performance traits, and n = 10 for the other antioxidant indicators and immunological parameters). The mean differences were tested at 0.05 level of significance using the post hoc Duncan’s test.

## 3. Results

### 3.1. Productive Performance

The effect of dietary supplementations of PR, BP, and their interaction on the broiler performance is shown in Table 3. The broiler productive performances from 1–21 d of age were nearly similar between groups and within the normal ranges of the Cobb-500 broiler’s performance guideline. From 22–42 d of age, PR and BP treatments substantially (*p* < 0.05) increased the FBW, BWG, and FI of broilers. In contrast, no significant differences were obtained in the FCR among PR or BP groups (*p* > 0.05). No interaction effect was observed in the performance traits of broilers except for the BWG, indicating the highest BWG in PR + BP combination group followed by PR, BP, and CONT group, respectively.

### 3.2. Antioxidant Indicators

The effect of PR, BP, and their interaction treatments on the plasma antioxidant indicators of broiler chickens is represented in Table 4. A significant increase in the TAC, T-SOD, and CAT activity was obtained in broilers treated with PR or BP. There was a significant interaction effect between PR and BP. In comparison with the CONT, the TAC, T-SOD, and CAT were significantly (*p* < 0.05) higher when PR or BP was added to the broiler diets, especially in the PR + BP group (*p* < 0.05).

### 3.3. Immunological Parameters

#### 3.3.1. Leukocytes Count, Differentiation, and Viability

The effect of PR, BP, and their interaction on the TWBC count, H/L ratio, and leukocyte cell viability of broiler chickens are presented in Table 5. TWBC was significantly (*p* < 0.05) increased by the PR treatment. The addition of PR or BP to broiler diets significantly (*p* < 0.05) decreased the H/L ratio and increased the LCV. There were no interaction effects for PR and BP on TWBC and H/L ratio. In contrast, the LCV was substantially (*p* < 0.05) enhanced by 13% and 10% in the PR and BP groups, respectively, while the highest level of LCV (18%) was obtained in the PR + BP combination group, compared to the CONT group. 

#### 3.3.2. Lymphocyte Proliferation and Humoral and Cellular Immunity

The results of lymphocyte proliferation and humoral and cellular immunity of broilers as affected by PR, BP, and their interaction are shown in Table 6. The addition of PR or BP into broiler diets significantly(*p* < 0.05) improved the T- and B-lymphocyte SI, SRBC-AB titer, and PHA-wattle immune reaction test. Except for B-lymphocyte proliferation, there was a significant interaction effect on the other traits (*p* < 0.05). The T-lymphocyte SI was elevated in the PR and BP groups compared to the CONT group by 40% and 53%, respectively, while it was elevated by 66% in broilers treated with PR + BP in combination. The broilers treated with PR, BP, or PR + BP exhibited a significant (*p* < 0.05) rise in the anti-SRBC-AB titer by approximately 16% than that in the CONT group. In the PHA-reaction test, the wattle thickness in broilers treated with PR or BP alone was significantly (*p* < 0.05) incremented by approximately 0.09 and 0.11 mm in comparison with the CONT broilers, while the highest wattle swelling (0.23 mm thicker than CONT) occurred in the broilers treated with PR + BP together.

#### 3.3.3. Immunoglobulin Assay

The effect of PR, BP, and their interaction on the immunoglobulin concentration in broilers is summarized in Table 7. A significant increase in the IgA, IgM, and IgG concentrations were obtained in the broilers treated with PR. The BP treatment significantly increased the levels of IgA and IgM in broilers. There was an interaction effect of PR and BP on the IgA concentration, showing the highest IgA levels in broilers treated with PR + BP in combination compared to the other groups. 

## 4. Discussion

There is a global consent that feeding antibiotics as growth promoters in livestock production can negatively affect human and animal health in the long run. Hence, seeking natural alternatives has been widely welcomed in the feed industry and research, especially in the field of poultry production [1]. In the present study, PR and BP were supplemented into broiler diets during 22–42 d of age at 1 g/kg either alone or in combination, and the results were investigated. The PR and BP levels applied in the previous studies were in a range between 25–5000 mg of PR and 300–45,000 mg of BP per kg of broiler diets [2,13,18,29]. The levels applied in the current work were justified according to a preliminary study, which include a diet with PR at a range of 0.025–5.0 g and BP at a range of 0.3–4.5 g per kg. Based on the results of the preliminary study, 1 g/kg of PR or BP into broiler diets was chosen considering the physiological effects and the economical prospective. 

Results show that dietary supplementation of PR or BP at a dose of 1 g per kg could improve some traits of the broiler performance such as FBW and BWG. These results, along with other reports [2], indicate that PR and BP could be successfully applied in broiler diets as natural growth promoters. The significant improvement in BWG of broilers treated with PR + BP, compared to the CONT and other groups, may be due to the nutritive value of PR and BP as additional sources of protein, lipids, and carbohydrates (Table 1). Similar results were also confirmed in broilers [17,30] and in other poultry species, such as turkey [14] and quail [15,18], and in rabbits [8]. In contrast, there is no obvious interaction effect between PR and BP on the FI and FCR of broilers in the present study. Moreover, Attia et al. [2] recorded a reduction in FI in the broiler groups supplemented from 0–35 d of age with 300 mg/kg diets of PR, PB, or their combination compared to the control broilers. Based on the relative weights of body organs (gizzard, liver, heart, intestine, pancreas, and abdominal fat) measured in their study, Attia et al. [2] concluded that reduced FI in PR- and BP-treated groups did not affect the development of the gastrointestinal tract.

As shown in Table 1, PR and BP contain many components with antioxidant activity, such as phenolic acids and flavonoids, and both have scavenging activity against DPPH-free radicals of approximately 85–90%. Compared with the CONT birds, the plasma TAC T-SOD and CAT levels were significantly increased by at least 30%, 81%, and3%, respectively, when PR and BP were added separately or in combination to the broiler diets (Table 4). The improvement of antioxidant defense system in broilers treated with PR and BP may be attributed to the direct capability of polyphenols and flavonoids that existed in the honeybee products to eliminate free radicals [2,31,32,33]. However, CAT activity seems to be less affected by PR and/or BP in the present study. This could be explained by the finding that the role of CAT enzyme as an antioxidant becomes more substantial during the high levels of oxidative stress [34], which did not occur in the present study. 

Results of several immunological parameters in broilers fed with PR and BP are intrinsically discussed in the current study. It was found that TWBC count increased, while H/L ratio decreased in broilers treated with individual PR or BP treatment. Similarly, Attia et al. [35] indicated that PR and/or BP supplemented at 300 mg/kg to Arbor Acers broiler diets significantly (*p* < 0.05) increased TWBC and decreased H/L ratio at 36 d of age compared to the control broilers. In contrast, the PR and BP treatments increased the leukocyte cell viability by 13% and 10%, respectively, compared to the control–untreated birds; moreover, this increment reached 18% over the control when PR and BP were supplemented in combination into broiler diets (Table 5). Similar to the results of the current study, a 40% increment in the leukocyte viability was demonstrated earlier in laying hens treated with PR compared to the untreated birds [25]. It was also suggested that antioxidant properties of propolis can contribute to the increasing leukocyte cell viability through the control of the fork-head box (Foxo) genes pathway involved in cellular apoptosis and oxidative stress resistance [36,37].

Compared to the CONT group, the lymphocyte proliferation was improved in broiler groups fed with PR or BP separately, while the maximum stimulation in T-lymphocytes was obtained in the PR + BP broiler group (Table 6). It was reported that immunomodulatory properties of PR and BP are associated with its high contents of flavonoids and phenolic acids [14,35,38]. Our results are in line with the documented findings that the bioactive and antioxidant compounds in the honeybee products, including PR and BP, sustain the thymus and bursa tissues to consequently generate active T and B lymphocyte cells, respectively, and augment the immune modulation via influencing the lymphocyte proliferation in birds [15,39,40]. In addition, B-lymphocytes are responsible for processing and presenting natural immunoglobulin antibodies [41]. It is known that introducing bee products into birds’ nutrition stimulates immuno-competence and triggers antibody production [18]. 

The present study also proved the beneficial effects of PR and BP on the humeral and cellular immunity in broilers. Results display remarkable increases in the antibody titer against SRBC and wattle swelling against PHA in those broilers supplemented with PR and/or BP (Table 6). In line with our results, the humeral and cellular immune responses in Japanese quail were enhanced in birds supplemented with ethanolic extract of PR or BP powder compared to the control birds [18]. The mechanisms underline these effects were slightly discussed in the literature. The stimulation of humoral immunity in the PR and BP groups may be attributed to the redistribution of peripheral blood leukocytes towards an augmentation in the lymphocyte populations compared to the other components [42]. Other reports suggest that antioxidant properties of flavones and phenols, which exist in PR and BP, may inhibit the synthesis of immunosuppressor, prostaglandin, and thus contribute to a higher humoral response [43]. It is also possible that these compounds prompt macrophages and lymphocytes to release interleukins, such as IL-1 and IL-2, which enhance the T- and B-cells’ mitogenesis [44]. Furthermore, PR and BP could stimulate the B-lymphocytes indirectly by increasing the anti-inflammatory cytokines then turning into plasma cells which in turn produce effective antibodies [45]. 

In a specific study on immunoglobulin titers against Newcastle vaccination in broilers [16], a significant increase in the IgM titer at 21 d of age was observed, but not in the IgG titer, in the BP-treated group vs. the control. In the current study, including PR or BP individually into broiler diets led to higher levels of IgM and IgA, which are partially considered as a natural, first-line defense in birds [46]. In contrast, there were no effects of PR and/or BP on the plasma IgM and IgG concentration, while a significant increase in IgA was obtained in the PR + BP group compared to the other groups (Table 7). These results agree with the fact assuming that IgM and IgG is more active after being challenged by infection [47]. 

## 5. Conclusions

Dietary supplementation with PR and BP separately or in combination could improve the growth performance and yield a higher body weight gain of broiler chickens. In addition, the total antioxidant capacity and superoxide dismutase activity were obviously increased in the treated broilers. Moreover, several immune functions were also enhanced by the PR and/or BP, such as leukocyte viability, lymphocyte proliferation, immunoglobulin concentration, and humoral and cellular immunity. Therefore, inclusion of PR and BP into broiler diets could be beneficial for broiler performance through improving the antioxidant and immune systems.

## Figures and Tables

**Table 1:** The chemical characteristics of propolis and bee pollen.

Item	Propolis	Bee Pollen
Dry matter (%)	90.8	90.5
Carbohydrate (g) ^1^	1.9 g	67.6
Crude fiber (g) ^1^	68.7	1.2
Total lipids (g) ^1^	9.2 g	3.7
Crude protein (g) ^1^	2.6 g	17.1
Total ash (g) ^1^	0.9 g	2.9
Phenolic content (mg GAE/g) ^2^	2.8	2.4
Flavonoid content (mg CAT/g) ^2^	1.4	0.9
DPPH-free radical scavenging activity (%) ^3^	89.3	84.5

^1^ Results of chemical analyses calculated per 100 g dry matter. ^2^ Calculated as mg gallic acid equivalent (GAE) or catechol equivalent (CAT), respectively, per g dry weight of the sample. ^3^ Calculated as % of the scavenging activity against 2,2-diphenyl-1-picrylhydrazyl (DPPH) radicals.

**Table 2:** The chemical characteristics of propolis and bee pollen.

Ingredients (g/kg)	Starter (0–8 d)	Grower (9–21 d)	Finisher (22–42 d)
Corn	607.0	654.0	693.0
Gluten meal	70.0	50.0	50.0
Soybean meal, 48% CP	289.0	243.0	203.0
Soybean oil	0.0	20.0	22.0
Di-calcium phosphate	4.0	4.0	4.0
Limestone	20.0	19.0	18.0
Salt	4.5	4.5	4.5
Vitamin–Mineral Premix ^1^	5.5	5.5	5.5
Nutritional composition			
Dry matter (g/kg) ^2^	906.0	901.0	908.9
Total ash (g/kg) ^2^	55.0	53.0	39.1
Crude protein (g/kg) ^2^	229.8	199.8	184.6
Crude fat (g/kg) ^2^	58.3	77.5	83.4
Crude fiber (g/kg) ^2^	32.0	35.0	35.8
Metabolizable energy (MJ/kg) ^3^	12.6	13.1	13.3
L-lysine (g/kg) ^3^	12.1	11.6	10.4
DL-Methionine (g/kg) ^3^	4.8	4.7	4.3
Calcium (g/kg) ^3^	9.1	8.6	8.1
Available phosphorus (g/kg) ^3^	4.5	4.2	4.1

^1^ Premix provides the following components per kg of the basal diet: vitamins A 10 KIU, D_3_ 5 KIU, E 65 IU, K 3 mg, B_1_ 3 mg, B_2_ 9 mg, B_6_ 4 mg, B_12_ 0.02 mg, biotin 0.20 mg, niacin 20 mg, pantothenic acid 15 mg, folic acid 2 mg, and choline chloride 500 mg; and minerals Mn 100 mg, Fe 40 mg, Zn 100 mg, Cu 15 mg, Se 0.35 mg, and Iodine 1 mg. ^2^ Determined values. ^3^ Calculated values.

**Table 3:** Effect of propolis (PR), bee pollen (BP), and their interaction (PR + BP) on the productive performance of broiler chickens.

Groups		Traits ^1^				
		IBW (g)	FBW (g)	BWG (g)	FI (g)	FCR
PR effect	−PR	755	2489 ^b^	1734 ^b^	154 ^b^	1.86
	+PR	745	2666 ^a^	1920 ^a^	165 ^a^	1.81
	n	10	10	10	10	10
	SEM	7.8	16.4	8.9	2.4	0.021
	*p*-value	0.376	<0.001	<0.001	0.003	0.095
BP effect	−BP	754	2528 ^b^	1774 ^b^	155 ^b^	1.84
	+BP	746	2628 ^a^	1881 ^a^	164 ^a^	1.83
	n	10	10	10	10	10
	SEM	7.8	16.4	8.9	2.4	0.021
	*p*-value	0.476	0.001	<0.001	0.024	0.669
Interaction effect	CONT	760	2421	1661 ^d^	148	1.87
	PR	749	2636	1887 ^b^	163	1.82
	BP	751	2559	1808 ^c^	160	1.86
	PR + BP	742	2697	1955 ^a^	168	1.80
	n	5	5	5	5	5
	SEM	10.9	23.1	12.5	3.4	0.030
	*p*-value	0.900	0.118	0.006	0.295	0.921

Means with dis-similar superscripts (a, b, c, d) in the same column significantly differ at *p* < 0.05. SEM: standard error of means. n: number of observations in the treatment group. ^1^ Traits: IBW, initial body weight 22 d of age; FBW, final body weight at 42 d of age; BWG, body weight gain; FI, feed intake; and FCR, feed conversion ratio.

**Table 4:** Effect of propolis (PR), bee pollen (BP), and their interaction (PR + BP) on the antioxidant indicators of broiler chickens.

Groups		Traits ^1^		
		TAC (U/mL)	T-SOD (U/mL)	CAT (U/mL)
PR effect	−PR	4.81 ^b^	4.57 ^b^	0.81 ^b^
	+PR	5.94 ^a^	6.70 ^a^	0.86 ^a^
	n	20	20	20
	SEM	0.046	0.114	0.009
	*p*-value	<0.001	<0.001	<0.001
BP effect	−BP	5.01 ^b^	4.33 ^b^	0.79 ^b^
	+BP	5.74 ^a^	6.95 ^a^	0.87 ^a^
	n	20	20	20
	SEM	0.046	0.114	0.009
	*p*-value	<0.001	<0.001	<0.001
Interaction effect	CONT	4.18 ^d^	3.08 ^d^	0.78 ^c^
	PR	5.84 ^b^	5.58 ^c^	0.80 ^bc^
	BP	5.43 ^c^	6.06 ^b^	0.83 ^b^
	PR + BP	6.04 ^a^	7.83 ^a^	0.91 ^a^
	n	10	10	10
	SEM	0.065	0.161	0.013
	*p*-value	<0.001	0.030	0.029

Means with dis-similar superscripts (a, b, c, d) in the same column significantly differ at *p* < 0.05. SEM: standard error of means. n: number of observations in the treatment group. ^1^ Traits: TAC, total antioxidant capacity; T-SOD, total superoxide dismutase; and CAT, catalase.

**Table 5:** Effect of propolis (PR), bee pollen (BP), and their interaction (PR + BP) on leukocytes count, differentiation, and viability of broiler chickens.

Groups		Traits ^1^		
		TWBC (10 ^3^/mL)	H/L Ratio	LCV (%)
PR effect	−PR	44.09 ^b^	0.43 ^a^	105 ^b^
	+PR	48.60 ^a^	0.38 ^b^	115 ^a^
	n	20	20	20
	SEM	0.455	0.005	0.4
	*p*-value	<0.001	<0.001	<0.001
BP effect	−BP	45.76	0.42 ^a^	106 ^b^
	+BP	46.93	0.39 ^b^	114 ^a^
	n	20	20	20
	SEM	0.455	0.005	0.4
	*p*-value	0.076	<0.001	<0.001
Interaction effect	CONT	43.24	0.45	100 ^d^
	PR	48.28	0.39	113 ^b^
	BP	44.94	0.41	110 ^c^
	PR + BP	48.93	0.37	118 ^a^
	n	10	10	10
	SEM	0.644	0.008	0.5
	*p*-value	0.422	0.194	<0.001

Means with dis-similar superscripts (a, b, c, d) in the same column significantly differ at *p* < 0.05. SEM: standard error of means. n: number of observations in the treatment group. ^1^ Traits: TWBC, total white blood cells; H/L ratio, heterophils to lymphocytes (H/L) ratio; and LCV, leukocyte cell viability.

**Table 6:** Effect of propolis (PR), bee pollen (BP), and their interaction (PR + BP) on lymphocyte proliferation and humoral and cellular immunity of broiler chickens.

Groups		Traits ^1^			
		T-lymphocytes SI	B-lymphocytes SI	SRBC-AB Titer (log2)	PHA-Wattle Test (mm)
PR effect	−PR	4.72 ^b^	2.27 ^b^	7.02 ^b^	0.51 ^b^
	+PR	5.70 ^a^	3.43 ^a^	7.51 ^a^	0.62 ^a^
	n	20	20	20	20
	SEM	0.034	0.031	0.046	0.003
	*p*-value	<0.001	<0.001	<0.001	<0.001
BP effect	−BP	4.47 ^b^	2.31 ^b^	7.06 ^b^	0.50 ^b^
	+BP	5.96 ^a^	3.39 ^a^	7.48 ^a^	0.63 ^a^
	n	20	20	20	20
	SEM	0.034	0.031	0.046	0.003
	*p*-value	<0.001	<0.001	<0.001	<0.001
Interaction effect	CONT	3.73 ^d^	1.71	6.51 ^b^	0.46 ^d^
	PR	5.21 ^c^	2.90	7.60 ^a^	0.55 ^c^
	BP	5.72 ^b^	2.83	7.53 ^a^	0.57 ^b^
	PR + BP	6.20 ^a^	3.95	7.43 ^a^	0.69 ^a^
	n	10	10	10	10
	SEM	0.048	0.043	0.065	0.004
	*p*-value	<0.001	0.500	<0.001	0.001

Means with dis-similar superscripts (a, b, c, d) in the same column significantly differ at *p* < 0.05. SEM: standard error of means. n: number of observations in the treatment group. ^1^ Traits: SI, stimulation index of T- and B-lymphocytes; SRBC-AB titer, sheep red blood cells antibody titer; and PHA-wattle test, phytohemagglutinin wattle test.

**Table 7:** Effect of propolis (PR), bee pollen (BP), and their interaction (PR + BP) on immunoglobulins of broiler chickens.

Groups		Traits ^1^		
		IgA (µg/mL)	IgM (µg/mL)	IgG (mg/mL)
PR effect	−PR	162.11 ^b^	440.48 ^b^	1.78 ^b^
	+PR	175.02 ^a^	502.62 ^a^	1.87 ^a^
	n	20	20	20
	SEM	0.490	2.426	0.042
	*p*-value	<0.001	<0.001	0.042
BP effect	−BP	162.97 ^b^	456.83 ^b^	1.85
	+BP	174.17 ^a^	486.27 ^a^	1.81
	n	20	20	20
	SEM	0.490	2.426	0.042
	*p*-value	<0.001	<0.001	0.506
Interaction effect	CONT	159.15 ^c^	427.57	1.84
	PR	166.79 ^b^	486.09	1.86
	BP	165.08 ^b^	453.39	1.73
	PR + BP	183.26 ^a^	519.16	1.89
	n	10	10	10
	SEM	0.693	3.430	0.060
	*p*-value	<0.001	0.298	0.248

Means with dis-similar superscripts (a, b, c, d) in the same column significantly differ at *p* < 0.05. SEM: standard error of means. n: number of observations in the treatment group. ^1^ Traits: IgA, immunoglobulin A; IgM, immunoglobulin M; and IgG, immunoglobulin G.

## Data Availability

The data presented in this study are available in the manuscript.

## Supplementary Materials

The following supporting information can be downloaded at: https://www.mdpi.com/article/10.3390/ani12131658/s1, Table S1: Antioxidants assay specification according to the colorimetric kits’ manufacturer; Table S2: Immunoglobulins (Ig) assay specification according to the ELISA kits’ manufacturer.
