# Supplementary material for: Modulation of Antioxidant Defense, Immune Response, and Growth Performance by Inclusion of Propolis and Bee Pollen into Broiler Diets
Source: Animals (Basel). 2022 Jun 28;12(13):1658. doi: 10.3390/ani12131658 (PMC9264778; doi:10.3390/ani12131658)
Supplement: Supplementary file 1 [file animals-12-01658-s001.zip › #Table S1.pdf]

Table S1. Antioxidants assay specification according to the colorimetric kits' manufacturer.

Technical parameter	TAC	T-SOD	CAT
Detection limits, U/mL	0.62 – 145.2	1.35 – 62.0	0.27 – 155.4
Sensitivity, U/mL	0.62	1.35	0.27
Intra-assay CV%	2.7	5.1	3.1
Inter-assay CV%	8.2	9.6	5.1

Abbreviations: TAC, total antioxidant capacity; T-SOD, total superoxide dismutase; CAT, catalase.
