# Supplementary material for: Modulation of Antioxidant Defense, Immune Response, and Growth Performance by Inclusion of Propolis and Bee Pollen into Broiler Diets
Source: Animals (Basel). 2022 Jun 28;12(13):1658. doi: 10.3390/ani12131658 (PMC9264778; doi:10.3390/ani12131658)
Supplement: Supplementary file 1 [file animals-12-01658-s001.zip › #Table S2.pdf]

Table S2. Immunoglobulins (Ig) assay specification according to the ELISA kits' manufacturer.

Technical parameter	IgA	IgM	IgG
Detection limits	12.5 – 400 ng/mL	7.8 – 2000 ng/mL	1.56 – 100 μg/mL
Sensitivity, U/mL	2.93 ng/mL	2 ng/mL	0.5 μg/mL
Intra-assay CV%	10	8	8
Inter-assay CV%	10	10	12
